# Perinatal Hypoxic-Ischemic Encephalopathy: epileptic and paretic outcome at one year of age

**DOI:** 10.1186/1824-7288-35-14

**Published:** 2009-06-04

**Authors:** Federico Allemand, Federica Reale, Marco Sposato, Alessandro Allemand

**Affiliations:** 1 Dipartimento di Scienze Neurologiche, Psichiatriche e Riabilitative dell'Età Evolutiva "Giovanni Bollea", "SAPIENZA" Università di Roma, Italia (Italy); 2 Dipartimento di Scienze Ginecologiche, Perinatologia e Puericultura, "SAPIENZA" Università di Roma, Italia (Italy)

## Abstract

**Background:**

The issue concerning neurologic outcome in patients with perinatal Hypoxic-Ischemic Encephalopathy (H.I.E) has inspired many studies which tried to identify adequate prognostic factors. Our work aims to find among neonatal parameters:

- factors which help to predict the risk to develop both Cerebral Palsy (CP) and secondary Epilepsy at one year of age in subjects affected by perinatal Hypoxic-Ischemic Encephalopathy,

- correlations between the neonatal parameters and the variable severity of above mentioned sequelae.

**Methods:**

We have recruited 32 subjects, whose history and neuroimages suggested a perinatal H.I.E and we have retrospectively analysed clinical-instrumental parameters at birth and at one year of age.

**Results:**

At one year cut-off, 9 patients developed both secondary epilepsy and CP (28%), whereas the other subjects showed only motor delay (31%), only secondary epilepsy (3%) or only CP (38%). Patients with both the severest sequelae were essentially term infants (only 2/9 were pre-term infants), with normal weight (only 3 LBW) and 7 of them with early pathologic EEG and neuroimages pointing out cortex injuries (typical of term infants). A statistic analysis showed the following correlations: birth weight and global prognosis (χ^2 ^= 14,03; p = 0,04); neonatal clinical pattern and CP's severity (χ^2 ^= 14,03; p = 0,0009); early EEG and CP's severity (χ^2 ^= 4,32; p = 0,04); epileptic onset age and CP and Epilepsy's severity (F = 16,01; p = 0,005).

Birth weight represented a predictive factor of early neurological outcome (<1,5 kg birth weight neonates are not at risk of both epilepsy and CP); neonatal clinical pattern and early EEG were correlated with variable severity of CP; an epileptic exordium in the first 6 months led up to a more severe epileptic and paretic outcome.

**Conclusion:**

From a clinical point of view it is of crucial importance to have some parameters which enable to discriminate patients at risk of more severe sequelae from those at risk of moderate severity outcome.

## Background

Perinatal Hypoxic-Ischemic Encephalopathy (H.I.E.), secondary to asphyxia, is one of the most important causes of acute perinatal mortality and neurological sequelae[1] which include severe disability (e.g. cerebral palsy, mental retardation, epilepsy) and clinical situations of moderate severity where the cerebral impairment establishes a neurodevelopmental delay, including for example a deficit of postural-motor acquirements or a reduced school performance.[2]

Our work aims to find:

- predictive factors concerning the risk of both Cerebral Palsy (C.P.) and Epilepsy, secondary to HIE, at one year of age. These factors should be searched among clinical-instrumental parameters registered at birth.

- Correlations between the clinical-instrumental parameters registered at birth and the variable severity of the above mentioned sequelae.

We suppose that having early prognostic factors for these important neurological sequelae of HIE should enable:

- the application of strategies to limit cerebral impairment by means of neuroprotection protocols[3];

- the application of early diagnosis protocols and the formulation of a rehabilitative and therapeutic strategy to improve the infants' quality of life [4,5].

## Methods

From June 2005 to June 2007 we have recruited 32 subjects between 1 and 8 years of age (among the patients attending our neurological Day Hospital^1^) with a clinical-instrumental diagnosis of HIE. The diagnosis was made on the basis of their general and neurological clinical picture and confirmed by cranial Ultrasonography and cerebral MRI images which showed hypoxic-ischemic cerebral impairments.

The exclusion criteria were:

- age below 1 year

- infective encephalopathy

- metabolic encephalopathy

- encephalopathy secondary to genetic or chromosomal diseases

- cerebral malformations

**Figure 1:**
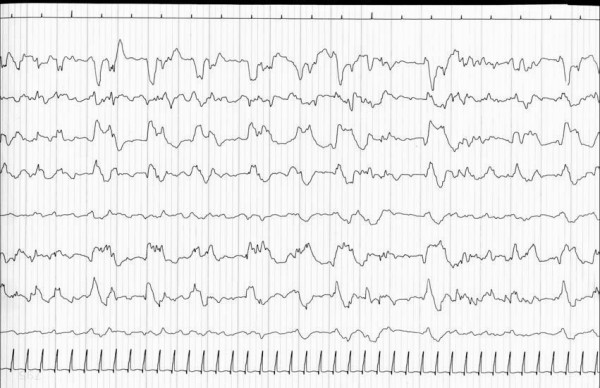
**electroencephalografical seizures and significantly altered EEG background rhythm in a two-day-old subject with diffuse post ischemic cerebral atrophy (Jasper montage)**.

The analysed parameters at birth were:

- gender: male (m) or female (f);

- gestational age (GA): term infants (t.i.) and preterm infants (p.i.), that is infants <37 weeks GA;

- type of delivery: spontaneous vaginal delivery (s.v.d.), cesarean section delivery (c.s.d), emergency cesarean section delivery (e.c.s.d.) due to pregnancy or labour complications which could endanger the mother's or foetus' health.

- birth weight: normal birth weight(NBW ≥ 2,5 kg), low birth weight (LBW ≥ 1,5–2,5 kg<), very low birth weight (VLBW ≥ 1–1,5 kg<), extremely low birth weight (ELBW < 1 kg);

- 5' Apgar Score (5'AS): infants were subdivided into <5 5'AS group and ≥ 5 5'AS group, this parameter plays not only an important role in the early clinical assessment of neonates at birth but it is also a significant prognosis factor[4,6];

- neonatal clinical pattern: infants with prevalent neurological symptoms attributable to Sarnat 2^nd^/3^rd ^stadium of HIE[6], infants with prevalent severe systemic phenomena of asphyxia (e.g. multiorgan involvement), infants with bland phenomena of asphyxia with neurologic symptoms attributable to Sarnat 1^st ^stadium of HIE[6];

- EEG characteristics at early hours/days (early EEG): this item is very important for the prognosis of neonates with HIE[4,7,8]; we have defined early EEG reports as pathological (presence of abnormalities in EEG background rhythm and/or presence of pathologic patterns) (Figure 1) and not pathological [9];

- type of encephalic hypoxic-ischemic injuries on the basis of cranial ultrasonography(US) reports at early hours/days of life: evidence of persistent periventricular hyperechogenicity (PVH), evidence of periventricular/intraventricular hemorrhage (PV-IVH), evidence of focal-multifocal parenchymal hyperechogenicity with/without cystic formation;

- type of encephalic hypoxic-ischemic injuries on the basis of MRI reports[1] at early hours/days of life: selective necrosis including lesions of basal ganglia (S.N.), parasagittal injury (P.S.I.), periventricular Leucomalacia(P.V.L.) and focal multifocal ischemic necrosis (F./M.I.N.).

At one year of age we have classified the patients on the basis of their neurologic outcome: we have identified patients with both secondary epilepsy and CP (which is one of the main objectives of our research), patients with only CP or secondary epilepsy and patients with only a transitory deficit of postural-motor acquirements (motor delay) with full recovery within the first few years of life.

Afterwards we have evaluated secondary epilepsy and CP severity.

Secondary epilepsy was classified as:

- highly severe: presence of partial/secondary generalized seizures, pluriweekly frequency of crisis, drug refractoriness of seizures, active or very active EEG pathological patterns;

- moderately severe: presence of partial seizures, 1 or <1 monthly frequency of crisis, good pharmacological control of seizures with crisis frequency reduction or disappearance, less active or absent EEG pathological patterns.

Furthermore we decided to register the epileptic onset age in patients with 2 severest sequelae (both CP and secondary epilepsy), supposing that it could be an useful parameter to better evaluate the outcome of the epileptic clinical picture.

CP was analyzed on the basis of neurologic examination, clinical observation and medical history in order to ascertain trunk control and sitting abilities within the first year of life [10].

This stage of psychomotor development is of crucial importance to determine the future prognosis of CP patients, especially in relation to their functional limitations (e.g. walking) and their performance in the various social contexts (home, school, community setting) [11,12].

CP was classified as:

- highly severe CP: at 1 year of life patients do not have trunk control and cannot maintain floor sitting.

- moderately severe CP: at 1 year of life patients have trunk control and can maintain floor sitting.

After collecting all data, we carried out a descriptive statistical analysis and a correlation study among the examined variables. The correlation study wanted to establish a statistical significance (indicated by a < 0,05 P-value) and it was carried out using the analysis of variance (ANOVA) for continuous variables and the M-L Chi Square Test for categorical variables.

## Results

The patient study group had the following characteristics:

- 20 m and 12 f;

- 19 t.i.(59%) and 13 p.i. (41%), average GA 35 weeks (range 24–41 weeks, median GA 39 weeks.);

- 18 NBW (56%), 6 LBW (19%), 2 VLBW(6%) and 6 ELBW(19%), average birth weight 2425 g (r. 680-4370 g, median BW 2615 g);

- 11 infants (34%) with 5'AS <5 (these subjects had signs of neonatal suffering due to severe or moderate asphyxia) and 21 infants (66%) with 5'AS ≥ 5.

- 12 s.v.d.(38%), 10 c.s.d.(31%) and 10 e.c.s.d. (31%)

- 10 patients (31%) with prevalent neurological symptoms (in particular neonatal seizures), 11 patients (34%) with prevalent severe systemic phenomena of asphyxia (essentially premature infants where the asphyxia was mainly expressed by symptoms such as sepsis, necrotizing enterocolite, respiratory distress etc...); 11 patients (34%) with bland phenomena of asphyxia (including neurological symptoms ascribable to Sarnat 1^st^stadium of HIE);

- 14 infants(44%) with normal and 18 patients (56%) with pathological early EEG;

- Pathological Cranial U.S in all 32 infants: PVH (38%), PV-IVH (16%), evidence of focal-multifocal parenchymal hyperechogenicity with/without cystic formation (47%);

- Pathological cerebral MRI in all 32 infants: S.N. (19%), P.S.I.(19%), P.V.L. (41%), F./M.I.N (22%);

The neurologic outcome within the first year of age showed the following results:

- 9 patients developed both secondary epilepsy and CP (28%) [see Additional file 1]: 7 were term infants (only 2 pre-term infants); 6 were NW (only 3 LBW); 7 with early pathologic EEG and neuroimages pointing out cortex injuries (typical of term infants[1]), these lesions are consistent with the generation of both an epileptogenical focus and a CP syndrome; the average age of epileptic onset was 5 months (median age 6 months) with 2 cases of neonatal exordium (neonatal seizures directly developed in a structured epileptic syndrome); 6 of these patients developed a highly severe epilepsy and a neuromotor clinical picture essentially characterized by highly severe CP, while 3 subjects developed a moderately severe epilepsy and a moderately severe CP.

- 10 patients developed only a motor delay (31%).

- 1 patient developed only secondary epilepsy, this infant was t.i., NBW with a ≥5 5'AS, with neonatal seizures and neuroimages indicating a P.S.I. and the patient manifested a moderately severe epilepsy at 1 year of age;

- 12 patients showed only CP (38%): 4 subjects developed highly severe CP and 8 developed moderately severe CP; 6 of these infants were T.I., 6 were P.T. and 7 were NBW with ≥5 5'AS.

It is worth to underline that all patients showing a moderately severe CP at one year of age, after their second birthday are able to walk, even if with some functional limitations, and do not need permanent assistive mobility devices. Whereas all patients showing a highly severe CP at one year of age, after their second birthday cannot walk autonomously, show important functional limitations and need assistive mobility devices.

These figures could find a correspondence with the GMFCS (Gross Motor Function Classification System), which was the assessment scale showing the best validity and clinical utility [11,12].

The statistic study showed the following correlations:

- birth weight and neurologic outcome (χ^2 ^= 14,03; p = 0,04)

- neonatal clinical pattern and CP severity (χ^2 ^= 14,03; p = 0,0009)

- early EEG and CP severity (χ^2 ^= 4,32; p = 0,04)

- epilepsy onset age and CP and Epilepsy severity (F = 16,01; p = 0,005)

## Discussion

**Figure 2:**
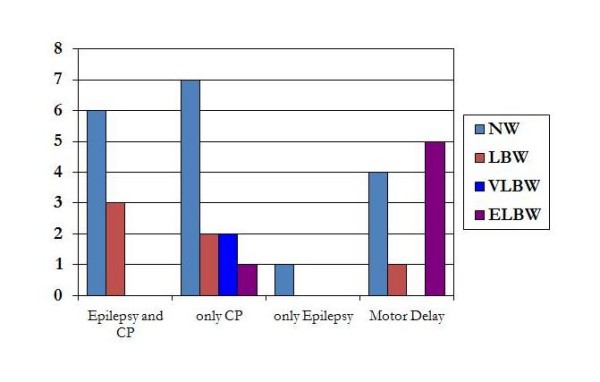
**birth weight and neurologic outcome**. < 1500 birth weight do not involve the risk of developing both CP and epilepsy within the first year of life.

Our study pointed out that birth weight is a predictive factor for neurologic outcome. We have observed that all ELBW and VLBW developed either CP or motor delay, therefore <1,5 kg birth weight neonates are not at risk of both epilepsy and CP at one year of age (Figure 2): in these subjects, typically preterm infants, the post-asphyxial injury involves essentially the white matter but also basal ganglia and thalamus[1], therefore it is possible to detect a neuromotor involvement compatible with a postural motor syndrome (such as cerebral palsy) but not compatible with an epileptic syndrome where the damage involves essentially the cerebral cortex[13].

**Figure 3:**
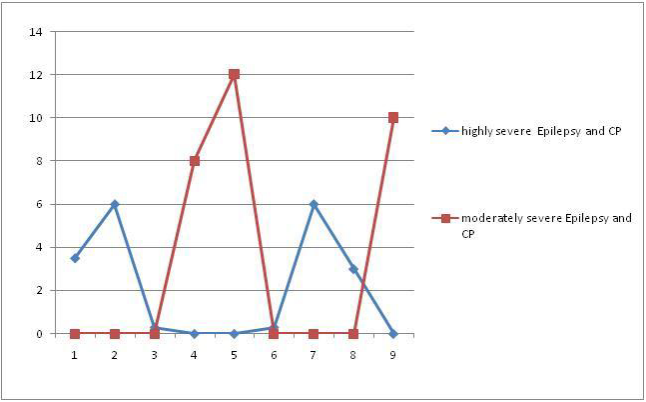
**Epilepsy onset within the first 6 months of life is an adverse prognosis factor for the neurologic outcome in patients with both epilepsy and CP within the first year of life**.

An epileptic onset within the first 6 months of age predicts a worse paretic and epileptic outcome (Figure 3): the patients were essentially term infants; neuroimaging revealed cortex injuries in most of them; at one year cut off they showed a highly severe epilepsy and CP clinical picture characterized by highly severe CP.

**Figure 4:**
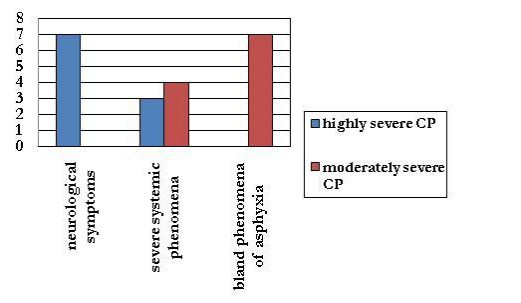
**neonatal clinical pattern and CP severity**. a severe neurologic pattern at birth is an adverse prognosis factor in terms of CP severity.

**Figure 5:**
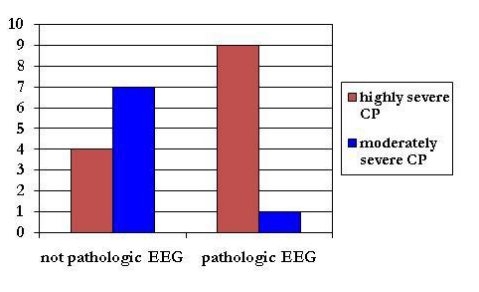
**early EEG and CP severity**. a pathologic early EEG is an adverse prognosis factor in terms of CP severity.

Neonatal clinical pattern or early EEG, as predictive factors of outcome, have been cited in many studies[4,5,7], which showed the key role of a major involvement of the central nervous system as a decisive factor for an abnormal outcome. The results of our study confirm these evidences (Figure 4, 5), indeed the presence of neurological symptoms ascribable to Sarnat 2^nd^/3^rd ^stadium of HIE[7], in particular neonatal seizures, and an early pathological neonatal EEG represent adverse prognostic factors in terms of CP severity: most of the subjects with highly severe CP, essentially term infants, have a typical neuropathological picture of very important lesions of cortex and/or basal ganglia, while most of the patients with moderately severe CP were preterm infants with bland phenomena of asphyxia at birth (with a neurological status ascribable to Sarnat 1st stadium of perinatal H.I.E.), white matter injury(sometimes also basal ganglia injury) and a not pathological early EEG.

## Conclusion

From a clinical point of view, it is of crucial importance to have some parameters which enable to discriminate patients at early risk of more severe sequelae from those at early risk of moderately severe outcome, in this way it is possible obtain a more manageable epilepsy and a CP susceptible of rehabilitation in order to improve the quality of life. This result could be achieved through the use of protocols for early diagnosis (e.g. including EEG and neurological examination follow up) or prevention (e.g. by the continuation or rational discontinuation of Anti-Epileptic Drugs therapy that was started at birth because of the presence of seizures).

Furthermore these results could be a starting point for a prospective study that includes a larger sample (considering the limited number of our patients) and a larger number of variables. Indeed our kind of approach, on the basis of more statistically significant data, could be for example very useful for the application of neuroprotection protocols in order to identify asphyxiated patients at risk of permanent encephalopathy[8] which could be prevented through selective cerebral hypothermia[3].

## Competing interests

The authors declare that they have no competing interests.

## Authors' contributions

AA dealt with the recruitment of case history. FR, MS, FA conceived the study, took part in the study planning and carried out the statistical analysis. All authors drafted the manuscript. All authors read and approved the final manuscript.

## Supplementary Material

Additional file 1 **Patients with both symptomatic epilepsy and cerebral palsy at one year of age**. Parameters at birth and at one year cut-off. Click here for file
