# Supplementary material for: Perinatal Hypoxic-Ischemic Encephalopathy: epileptic and paretic outcome at one year of age
Source: Ital J Pediatr. 2009 Jun 4;35:14. doi: 10.1186/1824-7288-35-14 (PMC2700127; doi:10.1186/1824-7288-35-14)
Supplement: Additional file 1 — Patients with both symptomatic epilepsy and cerebral palsy at one year of age. Parameters at birth and at one year cut-off. [file 1824-7288-35-14-S1.doc]

| **Pts.** | **GESTATIONAL AGE** | **BIRTH WEIGHT** | **TYPE**  **OF**  **DELIVERY** | **5 min**  **APGAR SCORE** | **NEONATAL**  **CLINICAL**  **PATTERN** | **EARLY NEONATAL**  **EEG** | **NEUROIMAGING** | **EPILEPTIC ONSET AGE** | **EPILEPSY**  **SEVERITY** | **CEREBRAL**  **PALSY**  **SEVERITY** |
| --- | --- | --- | --- | --- | --- | --- | --- | --- | --- | --- |
| **1** | **40 weeks** | **2630 gr** | **S.V.D.** | **8** | **Seizures** | **Pathologic** | **Parasagittal injury** | **3,5 months** | **High severity** | **High severity** |
| **2** | **40** | **3180** | **S.V.D.** | **4** | **Seizures** | **Pathologic** | **Selective necrosis** | **6** | **High severity** | **High severity** |
| **3** | **40** | **4370** | **E.C.S.D.** | **4** | **Status epilepticus** | **Pathologic** | **Multicystic encephalopathy** | **Neonatal** | **High severity** | **High severity** |
| **4** | **35** | **2400** | **C.S.D.** | **9** | **Bland phenomena of asphyxya** | **Not pathologic** | **Periventricular hemorragia** | **8** | **Moderate**  **severity** | **Moderate**  **severity** |
| **5** | **40** | **3380** | **C.S.D.** | **5** | **Bland phenomena of asphyxia** | **Pathologic** | **Selective necrosis** | **12** | **Moderate severity** | **Moderate**  **severity** |
| **6** | **39** | **2900** | **S.V.D.** | **8** | **Seizures** | **Pathologic** | **Selective necrosis** | **Neonatal** | **High severity** | **Moderate**  **severity** |
| **7** | **38** | **2424** | **S.V.D.** | **2** | **Severe systemic phenomena** | **Pathologic** | **Focal ischemic necrosis** | **6** | **High severity** | **High severity** |
| **8** | **40** | **3280** | **E.C.S.D.** | **7** | **Severe systemic phenomena** | **Not pathologic** | **Periventricular leucomalacia** | **3** | **High severity** | **High severity** |
| **9** | **33** | **1500** | **C.S.D.** | **4** | **Bland phenomena of asphyxya** | **Pathologic** | **Focal ischemic necrosis** | **10** | **Moderate severity** | **Moderate severity** |
